# Using variant databases for variant prioritization and to detect erroneous genotype-phenotype associations

**DOI:** 10.1186/s12859-017-1951-y

**Published:** 2017-12-01

**Authors:** Bart J. G. Broeckx, Luc Peelman, Jimmy H. Saunders, Dieter Deforce, Lieven Clement

**Affiliations:** 1 Laboratory of Animal Genetics, Faculty of Veterinary Medicine, Ghent University, Heidestraat 19, B-9820 Merelbeke, Belgium; 2 Department of Medical Imaging and Orthopedics, Faculty of Veterinary Medicine, Ghent University, Merelbeke, Belgium; 3 Laboratory of Pharmaceutical Biotechnology, Faculty of Pharmaceutical Sciences, Ghent University, Ghent, Belgium; 4 Department of Applied Mathematics, Computer Science and Statistics, Faculty of Sciences, Ghent University, Ghent, Belgium

**Keywords:** 1000 Genomes project variant database, Allele frequency, dbSNP, HapMap, Variant filtering, Variant database

## Abstract

**Background:**

In the search for novel causal mutations, public and/or private variant databases are nearly always used to facilitate the search as they result in a massive reduction of putative variants in one step. Practically, variant filtering is often done by either using all variants from the variant database (called the absence-approach, i.e. it is assumed that disease-causing variants do not reside in variant databases) or by using the subset of variants with an allelic frequency > 1% (called the 1%-approach). We investigate the validity of these two approaches in terms of false negatives (the true disease-causing variant does not pass all filters) and false positives (a harmless mutation passes all filters and is erroneously retained in the list of putative disease-causing variants) and compare it with an novel approach which we named the quantile-based approach. This approach applies variable instead of static frequency thresholds and the calculation of these thresholds is based on prior knowledge of disease prevalence, inheritance models, database size and database characteristics.

**Results:**

Based on real-life data, we demonstrate that the quantile-based approach outperforms the absence-approach in terms of false negatives. At the same time, this quantile-based approach deals more appropriately with the variable allele frequencies of disease-causing alleles in variant databases relative to the 1%-approach and as such allows a better control of the number of false positives.

We also introduce an alternative application for variant database usage and the quantile-based approach. If disease-causing variants in variant databases deviate substantially from theoretical expectancies calculated with the quantile-based approach, their association between genotype and phenotype had to be reconsidered in 12 out of 13 cases.

**Conclusions:**

We developed a novel method and demonstrated that this so-called quantile-based approach is a highly suitable method for variant filtering. In addition, the quantile-based approach can also be used for variant flagging. For user friendliness, lookup tables and easy-to-use R calculators are provided.

**Electronic supplementary material:**

The online version of this article (doi: 10.1186/s12859-017-1951-y) contains supplementary material, which is available to authorized users.

## Background

The identification of genetic variation responsible for a phenotype, is one of the key aims in the field of genetics. Already in 1999, the importance of a central variant database to facilitate the discovery of variant-phenotype associations was recognized and led subsequently to the establishment of dbSNP [1]. More recently, large scale projects like HapMap and the 1000 Genomes project variant database (1000G) were initiated that actively collect and sequence samples in order to identify the vast majority of genetic variation segregating in several populations [2, 3].

While these three databases all are publicly available variant catalogs, they differ in terms of scope, inclusion criteria and data collection [2–4]. For dbSNP, the scope of the database was “to provide a dense catalog of variants” and this catalog encompasses both “disease-causing clinical mutations and neutral polymorphisms” [1]. In addition, anyone can contribute to the database. For HapMap and 1000G, only samples fulfilling specific inclusion criteria (i.e. individuals that wanted to participate had to be legally competent adult donors that gave their explicit consent) were allowed and data was actively generated from a limited number of participating centers [2, 3]. These differences between databases can also influence the variants that end up in these databases. For example, based on the inclusion criteria of the latter two databases, for specific phenotypes, severely diseased individuals, are less likely to be included in the latter two databases.

Irrespective of the differences between these databases, they are often used the same way. Practically, this means that the facilitating role of variant databases for causal variant discovery lies currently mainly in reducing the large list of putative variants discovered during sequencing. This is often done according to one of the following approaches. The first approach is based on the assumption that disease-causing variants cannot reside in these databases [5, 6], hence, all variants in the variant database are used for filtering the list of putative disease-causing variants discovered during sequencing (called the “absence-approach”). The second approach employs allele frequency based cut-offs that differ with the mode of inheritance: for autosomal recessive (AR) disorders, all variants in databases with an allelic frequency of >1% can be used, while for autosomal dominant (AD) disorders, the threshold is set at 0.1% (called the “1%-approach”) [6]. As a consequence, a subset of the total number of variants inside these variant databases is used at that moment for filtering.

Even though the absence- and 1%-approach are part of the standard toolbox, to our knowledge, their performance has not been assessed yet. As such, the first aim was to assess the validity of these two standard approaches and at the same time compare their performance with a novel approach, the so-called quantile-based approach, which we believed would improve the search for novel variants responsible for Mendelian disorders. This quantile-based approach also applies frequency thresholds, but instead of static thresholds, these thresholds are variable and use prior knowledge on disease prevalence and inheritance data on one hand and database size and database characteristics on the other hand.

In addition, while databases are typically used for variant filtering, we also wanted to introduce an alternative application based of the quantile-based approach. This alternative application is based on the hypothesis that if a mutation is linked to a disease according to some proposed mode of inheritance, we should be able to predict its allelic frequency in a variant database. If this not the case, i.e. if the actual allelic frequency deviates sufficiently from these theoretical expectations, this might indicate that the proposed role of that variant in the disease has to be reconsidered.

## Methods

**Fig. 1:**
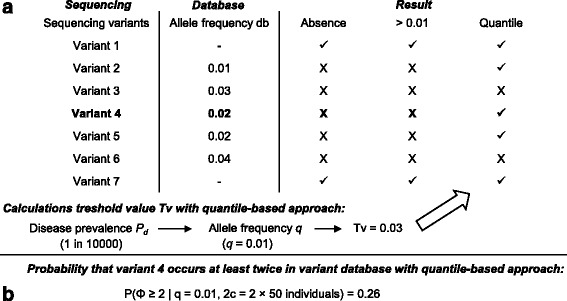
An overview of variant filtering (method 1) and variant flagging (method 2). A. method 1: In a sequencing study, a hypothetical list of 7 variants was discovered, with variant 4 being the causal variant and the other ones harmless co-inherited mutations. Inside the variant database, 5 out of 7 variants discovered during sequencing (including variant 4) are already represented with varying allele frequencies *f* (allele frequency db-column). Three different approaches for variant filtering can be used. Candidate variants that are filtered out, are denoted with an **X**. Candidate variants that are retained after filtering are denoted with a ✓. By assuming absence of disease-causing variants from variant databases (absence-approach), the disease-causing variant was erroneously filtered out. The same issue was encountered by using a static 1% threshold. The quantile-based approach was used to calculate a suitable allelic frequency threshold Tv. Based on the disease prevalence *P*_*d*_ of 1 in 10,000 individuals and an autosomal recessive mode of inheritance, the population allele frequency *q* is 0.01. For a variant database of 50 individuals (= 100 chromosomes, situation a), the Tv associated with the 95th quantile equals 0.03 (3/100). While the allele frequency *f* of the disease-causing variant in the variant database (= 0.02) is slightly higher than the theoretically expected population allele frequency (= 0.01) due to sampling variability, the Tv cut-off (0.03) has made it possible to discover the true disease-causing variant, while this was not the case for the other two approaches. B. method 2: this analysis determines how likely it is that a disease-causing variant (variant 4) occurs at least twice in a variant database of 50 individuals (= 100 chromosomes, situation a), given *P*_*d*_ equals 1 in 10,000 and an autosomal mode of inheritance. Based on the binomial distribution, this probability equals 0.26. As such, there is insufficient evidence to conclude that this model is inappropriate

In this section, we will first introduce the quantile-based approach. Next, two different methods for variant database usage based on this quantile-based approach are developed (Fig. 1). The aim of the first method is to provide an improved method for variant filtering when novel disease-causing variants are looked for (method 1). The starting point of this method is thus a large list of variants discovered during a new sequencing experiment that needs to reduced as much as possible. The second method uses the allele frequency of disease-causing variants that reside in variant databases to assess the validity of the proposed models. As such, this method allows the flagging of previously identified disease-causing variants that warrant additional research (method 2). The starting point of this method is thus previously discovered variants in variant databases for which an association with a phenotype has been reported.

### Terminology

Notation wise, the allelic frequencies for one locus are defined as *p* = frequency of allele 1 (A_1_), which is considered to be the normal wild type allele and *q* = frequency of allele 2 (A_2_), which is considered to be the deleterious mutant allele with *p* + *q = 1*. Based on Hardy-Weinberg equilibrium (HWE), the following relation exists between the aforementioned allelic frequencies and the corresponding genotype frequencies for the genotypes A_1_A_1_, A_1_A_2_ and A_2_A_2_: *P + H + Q = p* ^*2*^ *+ 2pq + q* ^*2*^ *= 1*.

Penetrance and detectance are defined as *P*_*pt*_ *= P (phenotype|genotype)* and *P*_*dt*_ *= P (genotype|phenotype)*, respectively [5, 7, 8]. Whereas disease prevalence *P*_*d*_ and the previously introduced allele and genotype frequencies are population parameters, the frequency of the disease-causing allele in the variant database is a sample parameter and is referred to as *f* (Fig. 1).

### The quantile-based approach

The quantile-based approach is short for “the expected allele frequency theoretical quantile-based filtering approach” that combines knowledge on disease prevalence, mode of inheritance, database size and database characteristics to calculate the expected allele frequency of the disease-causing allele in variant databases.

### Assumptions

The following assumptions are used:

1. All causal variants are bi-allelic and in HWE.
2. The disease prevalence *P*_*d*_ is error-free and from the same population the individuals included in the database are sampled from.
3. Each individual included in the database is sequenced at a sufficient sequencing depth and with sufficient quality to avoid allelic drop-out and sequencing errors.
4. All individuals and alleles are sampled independent and identically distributed.

If genetic heterogeneity is present, the following simplification is made: if *k* loci are responsible for a phenotype, they are assumed to be mutually exclusive. The validity of this approximation is based on the observation that the probability that two unlinked loci occur in a disease-state in one individual is extremely small, as demonstrated in Additional file 1.

### Linking population disease prevalence with expected allele frequency in variant databases and allele frequency thresholds: a quantile-based approach

The starting point is knowledge on the disease prevalence *P*_*d*_ *.* Given assumptions 1 and 2, when the disease prevalence *P*_*d*_ is known, it can be linked with *q* with different formulas according to the mode of inheritance. Several formulas are necessary as specific adaptations need to be considered when penetrance and/or detectance is reduced (Additional file 2). In addition, due to the inclusion criteria for HapMap and 1000G, not every individual from the population is available for sampling [2, 3]. The reason is that demanding legal competence might result in the selective exclusion of diseased individuals from participating. As this results in a selective exclusion of disease-causing alleles (relative to the wild type alleles), this might influence *f*. As such, separate formulas have to be developed for the situation where individuals can be selected at random from the entire population (situation a) or when individuals can only be selected from the non-diseased part of the population (situation b).

For situation a, it can be shown that the relation between *q* and *P*_*d*_ for fully penetrant and non-heterogeneous AD and AR diseases, respectively equals to:

AD $$ q=1-\sqrt{1-{P}_d} $$

AR $$ q=\sqrt{P_d} $$

Based on this relation, it can be seen that for an identical *P*_*d*,_ the *q* for AD diseases is far lower compared to AR diseases (See Additional file 3). When the same disease characteristics apply, but the sampling situation is limited to situation b, the formulas have to be adapted. The derivation of the aforementioned formulas for situation a and additional formulas for genetic heterogeneity, reduced penetrance and a combination of both for situation a and b are shown in Additional file 2.

Once *q*, the mutant allele frequency for the population, is calculated given a population disease prevalence *P*_*d*_, one can calculate with a certain probability at which upper frequency the disease-causing variant would reside in the variant database. These calculations are based on the quantile function of the binomial distribution for a random variable Φ, that represents the number of times the mutant allele occurs in the variant database, with parameters *2c* and *q* or *q’* (situation a or b, respectively (see Additional file 2)). For diploid organisms, c is the database size, expressed as the number of individuals. The upper frequency, or so-called threshold value Tv, calculated with this method, will be used later on to select certain variants in the variant database. More specifically, only variants residing in the variant database at an allelic frequency strictly higher than this Tv can be used for filtering the list of putative disease-causing variants discovered during sequencing. In terms of probability, this quantile-based Tv corresponds to *P (Φ ≤ Tv)* and a priori, it has to be decided which probability that *Φ ≤ Tv* is considered to be sufficient. Throughout our analysis, a 95% quantile-based approach is used, i.e. *P (Φ ≤ Tv)* = 0.95 or conversely, *P (Φ > Tv)* = 0.05*.*

### Method 1: improved identification of novel disease-causing variants (“variant filtering”)

#### Problem setting

When searching for novel disease-causing mutations, due to the output of massive parallel sequencers, a large number of variants are discovered. As a consequence, it is difficult to discriminate the small number of causal mutations from the large amount of harmless mutations that are co-inherited. At that moment, variants inside variant databases are typically used to reduce this list of candidate variants as much as possible. In other words, the aim of variant database usage is to reduce the amount of false positives (i.e. a harmless mutation passes all filters and is erroneously retained in the list of putative disease-causing variants) as much as possible. At the same time, it has been argued and even demonstrated that variant databases might actually contain disease-causing variants, which is called variant database contamination [5, 6, 9]. If a disease-causing variant occurs in a variant database and that database is used next for filtering variants discovered during sequencing, the true disease-causing variant might be erroneously removed from the list of candidate variants and cannot be discovered anymore [6]. This is defined as a false negative: the true disease-causing variant does not pass all filters, is thus removed from the list of putative disease-causing variants and as such, the correct mutation cannot be identified. It is clear that an optimal balance has to be found in order to restrict the number of both false positives and false negatives as much as possible.

In the next section, the two standard methods for variant database usage (the 1%-approach and the absence-approach) and the novel quantile-based approach are compared in terms of false positives and false negatives.

#### Assessment of variant database contamination inside 1000G for 30 AR diseases.

Practically, the absence-approach, the 1%-approach and the quantile-based approach were first compared in terms of the probability of encountering false negatives. This was done by alphabetically selecting diseases from the Genetics Home Reference National Institutes of Health database, based on the following three inclusion criteria: 1/ an AR mode of inheritance, 2/ the disease prevalence *P*_*d*_ is known and 3/ genes have been found to be associated with the phenotype. Selection was continued up until a total of 30 diseases were retained. Next, the actual allelic frequencies *f* of the causal mutations in the 1000G [3] were obtained by searching ClinVar [10] (filters: pathologic, single nucleotide variant, insertion, deletion, followed by a manual check of the associated condition) for disease-causing mutations in the genes suggested by Genetics Home Reference.

For the quantile-based approach, the following methodology was adopted. If *P*_*d*_ was provided as a range, the highest prevalence was used. The Tv (95th quantile) was calculated based on the *P*_*d*_, by using the formula for an AR mode of inheritance, assuming situation b. Tv calculations were done under the assumption of no genetic heterogeneity (i.e. detectance of 100%) because the number of different loci is generally unknown a priori.

Next, the number of false negatives were counted for each method. A false negative occurs when the disease-causing variant occurs at any frequency *f* > 0 inside the variant database, at a frequency higher than 1% or at a frequency higher than the Tv, for the absence-approach, 1%-approach and quantile-based approach, respectively.

#### Evaluation of the effect of filtering method on the number of variants available for filtering

The second assessment aims to evaluate the false positive probability. Determining an exact false-positive probability is difficult however, given that it is a count based on the number of variants discovered during sequencing and the number retained after filtering. These numbers depend however on several factors (type and/or assumption of the other filters used, realized sequencing depth, number of samples, …) that are not solely related to the problem of variant database usage. As such, a different approach was chosen: an approach that looks at the question from the database perspective: how many variants inside the database occur at a frequency higher than 1% or the Tv, respectively for the 1%-approach and the quantile-based approach. By definition, for the absence-approach, the entire set of variants from the database is available all the time.

For this analysis, a random subset from 1000G was used (UCSC table browser, settings: genome: Human, group: Variation, track: 1000G Ph3 Vars) [3, 11]. An important assumption (assumption 2) introduced earlier on states that the population used for the variant database is the same as the population from which the disease prevalence was obtained. The 1000G contains several subpopulations however [3]. This has to be taken care of as a disease prevalence might vary between populations, and as such, the allelic frequency might differ as well. To fulfill the assumption that the disease prevalence and the population to sample from are the same, all variants that did not segregate in the (European) population were removed. In total, the subset of 1000G contained 100,000 variants.

For the quantile-based approach, the following methodology was used: as the Tv for the quantile-based approach varies with *P*_*d*_, the 95% quantile-based Tv was calculated for each step for a *P*_*d*_ that varied between 1/1000 to 1/100000 (in steps of 1000). For each prevalence step, the number of variants with an allelic frequency higher than the Tv was counted.

#### Example of the quantile-based approach for variant filtering

To exemplify the quantile-based approach practically, an additional example is given, based on Miller syndrome. Miller syndrome was used as it was the first successful application of whole exome sequencing [12].

### Method 2: flagging suspicious disease-causing variants (“variant flagging”)

For previously identified disease-causing variants that reside in variant databases, the probability that they are present in a variant database at a frequency *f* or higher can be calculated when the disease-prevalence, mode of inheritance, database size and *f* are given. This corresponds to *1-P (Φ < f |q,2c) = P (Φ ≥ f |q,2c)*. The 13 variants from the first analysis that deviated sufficiently (i.e. with a probability of ≤0.05) from the theoretical expectancy, based on the proposed disease model, were investigated in detail to identify potential reasons for their unexpected high allelic frequencies in the 1000G variant database by performing a literature search in PubMed. Literature was searched stepwise. First, evidence for a link between the phenotype and genotype was searched for. If a causal association was reported, next, a search was conducted to identify potential reasons for this abnormal high frequency.

Throughout the paper, all analysis and simulations were conducted in R (version 3.3.2) using R-studio (version 1.0.44). Variant descriptions were checked with Mutalyzer 2.0.24 [13].

## Results

### Method 1: improved identification of novel disease-causing variants

**Fig. 2:**
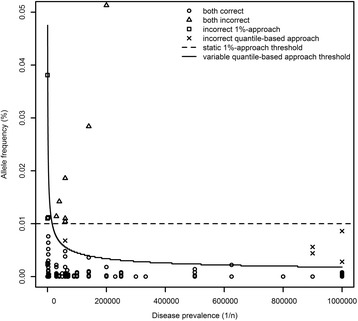
Actual allelic frequencies *f* of the disease-causing mutations for 30 autosomal recessive disorders. For a total of 1169 disease-causing mutations, the allelic frequency *f* was plotted, relative to the static 1% threshold and the variable quantile-based thresholds. For all variants, it was indicated whether they were correctly classified. Disease prevalence is expressed as 1/n (with n ranging from 0 to 1 000 000)

For the first analysis, 1169 mutations responsible for 30 AR diseases were identified. From the total number of mutations, 113 were found in 1000G (Fig. 2). For the absence-approach, this means that 113 disease-causing variants might not have been identified if the entire database is used for filtering the list of disease-causing variants. Expressed differently, the false negative probability was around 10% (113/1169) for the absence-approach. For the 1%-approach, the probability of false negatives was 1% (11/1169), i.e. eleven variants had an allelic frequency > 1%. The quantile-based approach resulted in a nearly identical false negative proportion of 1% as 13 out of 1169 variants had an allelic frequency higher than the 95th quantile Tv. When the list of false negative variants encountered with the 1% and the quantile-based approach were compared, 8 variants were found to be shared while 3 were uniquely missed by the 1%-approach and 5 by the quantile-based approach (Fig. 2). For the 1%-approach, the 3 unique false negative variants are associated with highly prevalent diseases, while for the quantile-based approach, 4 out of 5 unique false negatives are at the rare end of the spectrum.

**Fig. 3:**
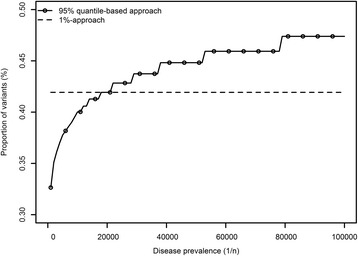
Relation between disease prevalence and proportion of the variant database available for filtering. The proposed mode of inheritance is autosomal recessive, the disease prevalence is expressed as 1/n (with n ranging from 1000 to 100,000). Both the variable quantile-based approach and the static 1%-approach are depicted. By definition, for the absence-approach all variants (100%) are available (not shown)

Next, the number of variants left in the database that can be used for variant filtering was evaluated for all three methods as an indirect method to estimate the false positives. Ideally, the number of false positives should be small, as such, the more variants that can be used for filtering, the better. By definition, for the absence-approach, 100% of the variants were available all the time. For the 95% quantile-based approach, the proportion of variants available ranged from 33% up to 47% for AR diseases for disease prevalences ranging from 1/1000 to 1/ 100,000 (Fig. 3). For the fixed 1%-approach, the number of variants remained fixed at the 42% level. While initially the quantile-based approach uses less variants from the database, as soon as *P*_*d*_ ≤ 1/18000, this changes: from that moment on, the quantile-based approach has more variants available for filtering.

#### Example for method 1

A detailed example is given for Miller syndrome, which is also known as postaxial acrofacial dysostosis or Genee-Wiedemann syndrome. Miller syndrome has an estimated prevalence of 1 in 1 million and was generally reported to be an AR disease, with one exception of AD inheritance [14, 15]. As such, an AR mode of inheritance was deemed most plausible and used for the analysis. As the 1000G was used for filtering, Tv calculations had to take into account that sampling was likely restricted to healthy individuals (situation b) [3]. A priori, it is generally unknown that cases typically have different mutations within the same gene, so a naïve analysis was conducted assuming no genetic heterogeneity.

From the total of 13 mutations currently identified [12, 16], 12 do not reside in the 1000 Genome variant database (Additional file 4). One mutation (rs201230446, Arg135Cys) does reside in the database, at *f* = 0.0004. Even though this disease is rare, variant database contamination has thus occurred. As a consequence, using all variants from the 1000G for filtering (the absence-approach) might result in a false negative. Based on the calculations, a naïve Tv of 0.0018 was found with the quantile-based approach. As *f* < Tv, applying this cut-off would thus not have resulted in a false negative and the same applies for the 1%-approach. In terms of false positives, 100%, 63% and 42% of the variant database would be available for filtering for the absence-approach, the quantile-based approach and the 1%-approach, respectively. The quantile-based approach thus outperforms the 1%-approach in terms of false positives, while it maintains a zero false negative probability for this specific disease.

### Method 2: flagging suspicious disease-causing variants

Based on the 95% quantile-based Tv, 13 variants occurred in the database at a frequency that was higher than expected (Additional file 5). These variants were thus flagged by the quantile-based approach to investigate their association with their respective phenotypes more deeply. For 2 variants, no evidence for any link with the phenotype was found. Two other variants were immediately considered to be normal polymorphisms upon discovery [17–19], while for one additional variant, the association with a phenotype was reported to be highly doubtful [20, 21]. For these 5 variants, it can thus be expected that they deviate from the theoretical values as they are not linked to the specified diseases.

For the 8 remaining variants, associations with their respective phenotypes were reported, as such, literature was searched for additional explanations for their unexpected high occurrence. For 7 variants, potential explanations were found. One variant was first classified as pathogenic, but no functional consequences were found later on, which subsequently resulted in a reclassification as a normal polymorphism [22, 23]. Other reasons were incorrect prevalence estimates (up to 20-fold higher [24, 25]), reduced penetrance [25], mild clinical phenotypes [19, 26] and incorrect modes of inheritances [19, 27–31] or a combination. In the end, for only 1 variant, no explanation for the high allelic frequency was found [32]. As such, 12 out of 13 variants were correctly flagged by the quantile-based approach as suspicious, based on their frequency *f* in 1000G. A detailed overview is presented in Additional file 5 for these variants.

## Discussion

When databases are used, quite often, no restrictions are made and all variants in these databases are used to filter the list of candidates [5, 12]. This so-called absence-approach works under the assumption that the specific disease-causing variant(s) for the studied disease can under no circumstances have been included in these databases. As a consequence, this also implies that the individuals that were used to construct the database, cannot at least carry one copy of the disease-causing mutant allele. Whether this is likely, depends firstly on the scope of the database, as introduced earlier on. Based on the inclusion criteria for HapMap and 1000G, severely diseased individuals might be excluded from these two databases [2, 3]. For dbSNP however, variant database contamination is far more likely. There are however several additional reasons why disease-causing variants can end up in variant databases [6, 9]. For AR diseases, all mutations are symptomless in a heterozygous state. Whenever penetrance is not complete, individuals that are genetically affected might not show it phenotypically. Some diseases are late-onset or the phenotype is actually very mild or might not even be considered to be a disease. Overall, there seem to be overt reasons to conclude that requiring a mutation to be absent is dangerous. This conclusion is also supported by our results as 10% false negatives were reported.

Taking the risk of variant database contamination into account, the static 1% or 0.1% approach has been suggested as an alternative [6]. This approach works as the false negatives decreased from 10% to 1% relative to the absence-approach. However, we demonstrated that this static threshold is too low for frequent AR disorders, while it is too high for rare AR disorders. While the former issue results in an increased probability to encounter false negatives, the latter can affect the number of false positives. This static approach is thus rather limited as it makes no use of epidemiologic data of the disease, neither of the characteristics or size of the database. One advantage is however that it is an easy rule of thumb, while our proposed models can require quite some calculations. To solve this, we provide look-up tables containing Tv cut-offs for AR and AD diseases for a wide range of disease prevalence and database sizes (Additional file 6-7) and also an R-script to calculate these cut-offs, together with sample code used to obtain the result for Miller syndrome (Additional file 8).

Overall, it is clear that no method is entirely error free. As such, an optimal balance has to be found in terms of both the false negative and the false positive probability. However, we do believe more priority should be given at a reduction of the false negatives. The reason is that the consequences of erroneously filtering out your actual disease-causing variant are irreversible, while the number of false positives can easily be reduced further by additional (filtering) steps [5, 7]. Examples of additional filters are eliminating variants that are not inherited in a manner compatible with the proposed mode of inheritance, sequencing additional affected individuals or controls and looking for shared/unshared mutations or genotypes and by removing synonymous variants [12]. Based on this weighting of false negatives relative to false positive errors, we consider the general absence-approach rather dangerous, especially now database sizes are increasing tremendously. A direct comparison of the quantile-based approach and the 1%-approach revealed a more or less identical false negative probability overall. Whereas the number of false positives will be slightly lower with the 1%-approach for highly prevalent diseases, the difference between the quantile-based approach and the 1%-approach decreases rapidly and as soon as the *P*_*d*_ drops beneath 1/18000, more variants will be available for filtering with the quantile-based approach, resulting in less false positives from that moment on. As such, while the false negative probability is nearly identical, it is likely that causal variant discrimination will be more easy with the quantile-based approach, especially for rare diseases.

The quantile-based approach proposed here is based on prior knowledge of both the disease studied and the database used. It starts with obtaining the correct disease prevalence *P*_*d*_ of the population and disease studied. When this has been done and the genetic disease characteristics have been determined, the theoretically expected mutant allele frequency *q* can be calculated with the appropriate formulas. In the final step, the appropriate Tv is calculated, based on the previously determined *q* and the database size. Clearly, the quantile-based approach is thus based on several assumptions. When the search is aimed towards the discovery of novel causal variants, it has to be assumed that this prior knowledge is correct.

The quantile-based approach can however also be used in a different way: a method to investigate the genotype-phenotype relation for “known” disease-causing variants that reside in variant databases and that have been reported to be associated with a phenotype previously. Contrary to the variant filtering method, this variant flagging method thus does not start from a list of variants that were discovered during a new sequencing experiment, but from variants that were previously discovered and reside inside a variant database. By comparing the theoretical disease characteristics with the actual database occurrence of variants, one can identify variants that do not follow these theoretical expectancies and even calculate the probability for this deviation to occur. It is important to stress that this method only allows flagging variants that warrant further attention: the exact cause of the deviations cannot be identified with the quantile-based approach. Based on the results, it is clear however that these variants are often flagged correctly and that deviations can be related to any of the assumptions.

A potential first cause for deviations and also the starting point for the quantile-based approach, is related to correct disease prevalence estimates. For variant filtering, if the disease prevalence estimate is an overestimation of the actual disease prevalence, *q* will be overestimated and accordingly the Tv will be set too high, hence less variants will be available for filtering leading to more false positives. The converse would happen if the prevalence is underestimated, as was the case for cerebrotendinous xanthomatosis (Additional file 5) where the Pro384Leu variant would have been filtered out erroneously [24, 25]. The same risk occurs when phenotypes are mild and as such can remain unrecognized, as was the case for one mutation associated with biotinidase deficiency. Precautionary measures to get the prevalence estimates correct are that estimates have to be based on a representative, sufficiently sized sample and clear, standardized diagnostic criteria should be used.

Secondly, the appropriate formulas to link *P*_*d*_ with *q* and calculate the Tv have to be used. As such, the correct mode of inheritance, penetrance and probability of genetic heterogeneity have to be chosen. Whereas genetic heterogeneity tends to reduce the individual allelic frequencies of disease-causing alleles, the opposite happens when the penetrance is incomplete. As such, where an unexpected genetic heterogeneity reduces the probability of encountering a false negative, an unexpected reduced penetrance, especially when reduced severely, increases this probability. Based on the examples and our focus on reducing false negatives, especially reduced penetrance is thus a potential issue. One example is again related to cerebrotendinous xanthomatosis where a reduced penetrance led to unexpectedly high *f* [24, 25]. The disease model can however also be incorrect, as discovered for ataxia with oculomotor apraxia and Bardet Biedl [27, 29].

From the database perspective, two other assumptions were made. Firstly, it was assumed that every individual was sequenced sufficiently in order to avoid allelic drop-out and, secondly, it was assumed that no sequencing errors occurred. The effect of sequencing errors can go in two directions: if a sequencing error occurs predominantly in the direction of the mutant allele, *f* might be (falsely) higher than expected based on the disease prevalence. If it is the other way around, *f* is lower. The latter would actually have no important consequence: the Tvs are not influenced by this (they are based on disease prevalence and database size), *f* will have even decreased and as such, the probability of being erroneously removed decreases as well. Linked to sequencing error is the sufficient sequencing/allelic drop-out assumption because in general, a higher sequencing coverage is associated with a reduced sequencing error and because the consequences are the same. Missing a mutant allele, results in a decreased *f*, hence nothing bad happens. The converse happens when a wild type allele is lost: *f* increases in the variant database, corresponding to an increased probability of erroneous removal of the disease-causing variant.

Two final causes for deviations are that the HWE assumption might be invalid and that deviations from the theoretical model can always occur by chance. While HWE can be tested for, by chance deviations and the database assumptions are difficult to evaluate. Either of the latter three assumptions might actually explain the high frequency of the Ile294Val variant for biotinidase deficiency as no other reason could be pinpointed [32].

While all assumptions were discussed separately, it is important to stress that several assumptions can be jointly incorrect. Even though the exact cause for deviations differs, their net consequences are the same: *f* occurs either at higher frequencies than expected or at lower frequencies than expected. In the case *f* occurs at lower frequencies than expected, the Tv could have been set lower and as such, the probability of encountering false positives might be slightly increased. As discussed earlier on, this is not necessarily an issue as the number of false positives can often be reduced further by additional (filtering) steps [5, 7]. Whenever *f* occurs at higher frequencies than expected, the potential consequences are far more dangerous as this results in an increased false negative probability. Based on the 1% false negative probability for the quantile-based approach which is based on real-life data, the quantile-based approach does seem to control this false negative probability relatively well. In addition, if a variant was missed, in 12 out of 13 times, it was actually correctly flagged as the correlation between genotype and phenotype did not follow the prespecified model.

Whereas the scope of this article is directed towards Mendelian disorders, it can be questioned whether the methodology proposed here might be of broader use, i.e. can it be used for complex disorders as well? For several reasons however, this is not necessarily the case. Complex disorders are the resultant of the combined effect of genetic and environmental factors and typically, this genetic contribution itself is due to the combined effect of several co-occurring variants [33, 34]. As such, the negligible probability that several mutations occur together is due to the definition of complex disorders invalid. This assumption is however necessary to link disease prevalence directly to the probability of observing a certain mutant allele for an individual locus. A potential solution is based on the general architecture of complex disorders [33]: assuming a *q* of 0.05 for common variants, the *P (Φ ≤ Tv)* can be calculated for one variant. The overall probability for *k* independent loci to occur ≤ Tv is the product of these individual probabilities. This is only an approximate solution however: it still requires an assumption on the number of loci and also assumes the disease follows the general architecture of complex disorders. As such, the proposed models might thus be of limited use for complex disorders.

## Conclusions

To conclude, we developed and evaluated a novel method for variant filtering, based on disease characteristics on one hand and database characteristics on the other hand. This so-called quantile-based approach has a similar false negative probability relative to the 1%-approach, but will often result in (far) less false positives. In addition, it can be used to correctly identify variants that deviate from their proposed role in specific phenotypes. For practical usability, R functions and tables are provided. Overall, we believe the quantile-based approach will lead to improved variant database usage in the search for novel disease-causing mutations and to assess phenotype-genotype relations.

## Additional files

Additional file 1: Prove that the probability that two unlinked loci in a disease-state are co-inherited is small. (DOCX 13 kb)

Additional file 2: The derivation of all the models. (DOCX 34 kb)

Additional file 3: Disease prevalence, genotype and allele frequencies for autosomal recessive (A) and dominant (B) disorders, respectively. Each graph depicts the mutant allele frequency *q,* the frequency of heterozygous genotypes (*H*) and homozygous mutant allele genotypes (*Q*) for a varying disease prevalence *P*_*d*_ of 1/n (with n ranging from 1000 to 100,000). For an autosomal recessive disease (A), the disease prevalence is equal to *Q*. For an autosomal dominant disease (B), the disease prevalence is equal to the sum of *Q* and *H*. Given the low contribution of Q relative to H, *P*_*d*_ is graphically nearly perfectly superimposed on *H*. (TIFF 613 kb)

Additional file 4: Variants associated with Miller syndrome and their allelic frequency in the 1000 Genomes variant database. (XLS 49 kb)

Additional file 5: Variants inside the variant database flagged by the quantile-based approach for further investigation. (XLS 42 kb)

Additional file 6: Allele frequency cut-offs for autosomal recessive disorders for various database sizes and disease prevalence. (XLS 73 kb)

Additional file 7: Allele frequency cut-offs for autosomal dominant disorders for various database sizes and disease prevalence. (XLS 60 kb)

Additional file 8: An r-script containing the “Tv_threshold” function for Tv cut-off calculation and the “flag_probability” function to calculate the probability that variants occur at least with a frequency f in the database, together with practical examples. (R 8 kb)

## Abbreviations

1000G: 1000 Genomes project variant database
AD: Autosomal dominant
AR: Autosomal recessive
HWE: Hardy-Weinberg equilibrium
Tv: Threshold value
