# Supplementary material for: Using variant databases for variant prioritization and to detect erroneous genotype-phenotype associations
Source: BMC Bioinformatics. 2017 Dec 1;18:535. doi: 10.1186/s12859-017-1951-y (PMC5710091; doi:10.1186/s12859-017-1951-y)
Supplement: Supplementary file 1 — Prove that the probability that two unlinked loci in a disease-state are co-inherited is small. (DOCX 13 kb) [file 12859_2017_1951_MOESM1_ESM.docx]

**Additional file 1: Prove that the probability that two unlinked loci in a disease-state are co-inherited is extremely small.**

The following simplification can be made for the detectance if the probability that two unlinked loci occur in a disease-state is negligible. Under genetic heterogeneity (i.e. reduced detectance): if *k* loci are responsible for a phenotype, they are assumed to be mutually exclusive, resulting in the following formula.

$$P_{1dt}+ P_{2dt}+{\ldots+ P}_{kdt}= \sum_{i=1}^{k} P_{idt}=1$$

The derivation is based on the rule for the sum of probabilities for event A or event B:

$$P\left( A \cup B \right)=P\left( A \right)+P\left( B \right)-P(A\cap B)$$

If $P\left( A\cap B \right)=0$, the sum simplifies to the proposed equation.

While it is an approximation, it can be shown that the probability of A and B occurring at the same time, is really small for both autosomal recessive (AR) and autosomal dominant (AD) diseases.

For an AR disease: what is the probability of, given a phenotype, observing the disease genotype for two independent loci (loci A and B, with respective genotype frequencies represented by $q_{A}^{^{2}}$ and $q_{B}^{^{2}}$) at the same time?

$$P\left( A \cap B \right|phenotype)= q_{A}^{^{2}} \times q_{B}^{^{2}}$$

When compared to the other terms in the basic probability rule, it is clear that it is far smaller than either of the first two terms ($q_{A}^{^{2}} +q_{B}^{^{2}})$.

For an AD disease, the situation is a bit more complex, given that :

$$P\left( A \cap B \right|phenotype)= {{2 p}_{A}q}_{A}\times{2p}_{B}q_{B}+q_{A}^{^{2}} \times q_{B}^{^{2}}+{{2 p}_{A}q}_{A}\times q_{B}^{^{2}}+{2p}_{B}q_{B}\times q_{A}^{^{2}}$$

The final three terms each time contain a product with a homozygous genotype and, as demonstrated in Supp. Figure 1, for AD diseases, homozygous genotypes are (very) rare. In this case, they are multiplied further, resulting in an even more extreme situation. The product of the two heterozygous loci is the biggest term out of these four, but also far smaller than the individual probability of heterozygous loci. Overall, it thus seems a reasonable approximation to use the following rule: $P\left( A \cup B \right)\approx P\left( A \right)+P\left( B \right)-P(A\cap B)$
