# Supplementary material for: Using variant databases for variant prioritization and to detect erroneous genotype-phenotype associations
Source: BMC Bioinformatics. 2017 Dec 1;18:535. doi: 10.1186/s12859-017-1951-y (PMC5710091; doi:10.1186/s12859-017-1951-y)
Supplement: Supplementary file 2 — The derivation of all the models. (DOCX 34 kb) [file 12859_2017_1951_MOESM2_ESM.docx]

**Additional file 2. The derivation of all the models developed for different sampling situations (a and b), genetic heterogeneity, reduced penetrance and a combination of both, for both autosomal recessive and autosomal dominant disorders.**

Situation a: at random selection from the entire population

The starting point is knowledge on the disease prevalence *P_d_.* Given the assumptions introduced in the main text, when the disease prevalence *P_d_* is known, it can be linked with *q* with different formulas according to the mode of inheritance. The aim of all subsections is always to find a formula for *q*, given a certain *P_d_*.

1. Autosomal dominant (AD)

Based on the definition of AD diseases, it is clear that the prevalence of AD diseases equals the sum of the individuals that are homo- and heterozygous for the mutant allele. Assuming full penetrance and no genetic heterogeneity, the following relation between phenotype and genotype can be made:

$$P_{d}=G_{AD}=Q+H=q^{2}+2pq=1-p^{2}$$

With:

P_d_ = the disease prevalence

G_AD_ = the (genetic) probability of AD disease

Rearranging this equation, *q* is equal to:

$$p^{2}=1-P_{d}$$

$$p=\sqrt{1-P_{d}}$$

$$1-q=\sqrt{1-P_{d}}$$

$$q=1-\sqrt{1-P_{d}}$$

1. Autosomal recessive (AR)

In this case, the prevalence is solely the result of the individuals that are homozygous for the mutant allele. Assuming full penetrance and no genetic heterogeneity, the following relation between phenotype and genotype can be made:

$$P_{d}= G_{AR}=Q=q^{2}$$

With:

G_AR_ = the (genetic) probability of AR disease

Rearranging the equation in function of *q*, gives the following result:

$$q= \sqrt{P_{d}}$$

1. Reduced penetrance

Reduced penetrance has the consequence that not every genetically affected individual is actually recognized as one. The consequence is that the phenotypical disease prevalence is actually an underestimation of the genetically affected population. Mathematically, assuming no genetic heterogeneity, this can be described as:

For AD diseases:

*(Formula 1)*

$$P_{d}=P_{ptQ} \times Q+P_{ptH} \times H={P_{ptQ} \times q}^{2}+P_{ptH} \times2pq$$

With:

P_ptQ_ = the penetrance of genotype Q

P_ptH_ = the penetrance of genotype H

This formula can be simplified if the penetrance is the same for the hetero- and homozygous genotype (i.e. $P_{ptQ}=P_{ptH}=P_{pt})$:

*(Formula 2)*

$$P_{d}=P_{ptQ} \times Q+P_{ptH} \times H=P_{pt} \times(Q+H)={P_{pt} \times(q}^{2}+2pq)$$

$$= P_{pt} \times(1-P)=P_{pt} \times(1-p^{2})$$

Rearranging both formulas to obtain a result for $q$ gives the following results:

Formula 1:

$$P_{d}={P_{ptQ} \times q}^{2}+P_{ptH} \times2q\left( 1-q \right)$$

$$\leftrightarrow0={P_{ptQ} \times q}^{2}+P_{ptH} \times2q\left( 1-q \right)-P_{d}$$

$$\leftrightarrow0={P_{ptQ} \times q}^{2}+2P_{ptH}\times q-2P_{ptH} \times q^{2}-P_{d}$$

$$\leftrightarrow0={(P_{ptQ}-2P_{ptH}) \times q}^{2}+{2P}_{ptH}\times q-P_{d}$$

If $P_{ptq}-2P_{pth} \neq0$, the solution for this equation is:

$$\Delta=b^{2}-4 ac=\left( 2\times P_{ptH} \right)^{2}-4 \times(P_{ptQ}-2P_{ptH})\times\left( -P_{d} \right)$$

If $\Delta> 0$, then:

$$q= \frac{(-2 \times P_{ptH})\pm\sqrt{\Delta}}{2 (P_{ptQ}-2P_{ptH})}$$

If $\Delta= 0$, then:

$$q= \frac{(-2 \times P_{ptH})}{2 (P_{ptQ}-2P_{ptH})}$$

Formula 2:

$$P_{d}= P_{pt}-p^{2}\times P_{pt}$$

$$p^{2}\times P_{pt}=P_{pt}-P_{d}$$

$$p= \sqrt{\frac{P_{pt}-P_{d}}{P_{pt}}}$$

$$1-q=\sqrt{\frac{P_{pt}-P_{d}}{P_{pt}}}$$

$$q=1- \sqrt{\frac{P_{pt}-P_{d}}{P_{pt}}}$$

If $P_{ptQ}=P_{ptH}$, the first and second formula are equivalent. In the R-calculator, the simplifying assumption of equal penetrance is used (formula 2).

For AR diseases:

$$P_{d}= P_{pt}\times G_{AR}=P_{pt}\times Q={P_{pt}\times q}^{2}$$

Rearranging the equation, gives the following result:

$$q= \sqrt{\frac{P_{d}}{P_{pt}}}$$

1. Genetic heterogeneity

Under full penetrance and based on the simplifying assumption for genetic heterogeneity, the following formulas apply:

For AD diseases, caused by *k* loci:

$$P_{d}=G_{1AD}+ G_{2AD}+\ldots+ G_{kAD} =\left( Q_{1}+H_{1} \right)+\left( Q_{2}+H_{2} \right)+\ldots+ \left( Q_{k}+H_{k} \right)$$

For AR diseases, caused by *k* loci:

$$P_{d}= G_{1AR}+G_{2AR}+\ldots{+G}_{kAR}=Q_{1}+Q_{2}+\ldots+Q_{k}$$

If the probability of detectance is known for each genotype, this formula resolves to estimating the mutant allele frequency for the individual genotypes, based on the definition that:

$$\sum_{i=1}^{k} P_{dti}=1$$

The relative contribution of each individual locus *i* is equal to:

$$P_{d}{\times P}_{dti}=G_{iAD} or P_{d}{\times P}_{dti}= G_{iAR}$$

Once the value of $G_{iAR}$ or $G_{iAD}$ is known, $q_{i}$ can be calculated based on the appropriate formulas introduced earlier.

1. Combination of genetic heterogeneity and reduced penetrance

In this case, the phenotype can be caused by several loci, each with a reduced (and potentially different) penetrance. For AD and AR with *k* loci, the following formulas should be used:

AD:

$$P_{d}={(P}_{1ptQ}\times Q_{1}+P_{1ptH}\times H_{1})+{(P}_{2ptQ}\times Q_{2}+P_{2ptH}\times H_{2})+\ldots+ {(P}_{kptQ}\times Q_{k}+P_{kptH} \times H_{k})$$

$$= \sum_{i=1}^{k} {(P}_{iptQ}\times Q_{i}+P_{iptH}\times H_{i})$$

AR:

$$P_{d}= {(P}_{1ptQ} \times G_{1AR})+{(P}_{2ptQ}\times G_{2AR})+\ldots{+ {(P}_{kptQ} \times G}_{kAR})$$

$$={(P}_{1ptQ}\times Q_{1})+ {(P}_{2ptQ}\times Q_{2})+ {\ldots+ (P}_{kptQ}\times Q_{k})$$

$$= \sum_{i=1}^{k} {(P}_{iptQ}\times Q_{i})$$

The relative contributions of the *k* contributing loci are calculated by multiplying *P_d_* with the respective probability of detectance *P_dt_* for each specific locus.

Situation b. Ad random selection amongst healthy individuals

For several reasons, it is not necessarily true that the entire population can be sampled: in some databases, diseased individuals are unlikely to contribute directly. As this affects the sampling population, this will also have an effect on the allele frequencies of mutant alleles in the database. To calculate the appropriate mutant allele frequency *q’* in the subpopulation, first, the appropriate formula from situation a has to be used to calculate *q*. Next, the correct formula from the following ones has to be used to calculate *q’*.

1. AD

For a fully penetrant, non-heterogeneous AD disease-causing mutation, every individual that at least carries one copy of the mutant allele is omitted. Hence, theoretically, only individuals with the $P$genotype (= $p^{2}$) remain for the sampling, which results in an absence of the mutant allele in the database.

1. AR

For a fully penetrant, non-heterogeneous AR disease-causing mutation, all $Q$ individuals (=$q^{2}$) show the phenotype and are thus omitted from the database. While the relation among disease prevalence and $q$ remains the same in the population, the probability of sampling $q$ has changed: the total population to sample from is not $P+H+Q$, but $P+H$. So stepwise, after $q$ has been calculated based on the general population (situation a.2), the mutant allele frequency has to be corrected to $q^{'}$ based on the following calculations:

Starting from *q*:

$$P=\left( 1-q \right)^{2}$$

$$H=2\times\left( 1-q \right)\times q$$

$$q^{'}=\frac{\frac{H}{2}}{P+H}=\frac{\frac{H}{2}}{1-Q}= \frac{\frac{H}{2}}{1-P_{d}}= \frac{q\times(1-q)}{1-P_{d}}$$

1. Reduced penetrance

Assuming no genetic heterogeneity, for AD, the denominator is in this case the sum of all healthy individuals combined with the non-diseased individuals that are homozygous or heterozygous for the mutant allele. For AR, the denominator is in this case the sum of all healthy individuals combined with the non-diseased individuals that are homozygous for the mutant allele. In both cases, this basically means that only the phenotypically healthy individuals can be sampled from, which equals the population minus the disease frequency.

For an AD disease:

$$q^{'}= \frac{(1-P_{ptH})\frac{H}{2}+(1-P_{ptQ})Q}{P+(1-P_{ptH})H+(1-P_{ptQ})Q}=\frac{(1-P_{ptH})\frac{H}{2}+(1-P_{ptQ})Q}{1-P_{d}}$$

As a control, if $P_{ptQ}{=P}_{ptH}=1$, $q^{'}$ = 0 as described in 2.b.a.

For an AR disease:

$$q^{'}= \frac{\frac{H}{2}+(1-P_{t})Q}{P+H+(1-P_{t})Q}= \frac{\frac{H}{2}+(1-P_{t})Q}{1-P_{d}}$$

The sampling population has thus increased from $P+H$ (situation b.2) to $P+H$ + the fraction of $Q$ that does not show the disease.

1. Genetic heterogeneity

For an AD disease: assuming full penetrance, again, only $p^{2}$ individuals remain for each individual locus. As a result, no single mutation should be in.

For an AR disease, again assuming full penetrance: for the calculation of $q_{i}^{'}$ in the subpopulation to be valid, removal of all the affected individuals can have no effect on the relative proportions of $H_{i}$and $P_{i}$individuals for each individual *i*th locus. Under the assumption of loci inheriting independently, the allelic frequencies in the subset of the population are the same as in the general population. The following formula is obtained:

$$q_{i}^{'}=\frac{\frac{H_{i}}{2}}{1-G_{iAR}}= \frac{\frac{H_{i}}{2}}{1-{(P}_{d}\times P_{dti})}$$

1. Combination of genetic heterogeneity and reduced penetrance

The equation for $q^{'}$ for the *i*th locus, assuming independent inheritance is:

For an AD disease:

$$q_{i}^{'}=\frac{(1-P_{iptH})\frac{H_{i}}{2}+(1-P_{iptQ})Q_{i}}{1- {(P}_{d}\times P_{dti})}$$

For an AR disease:

$$q_{i}^{'}=\frac{\frac{H_{i}}{2}+(1-P_{it})Q_{i}}{1- {(P}_{d}\times P_{dti})}$$
